# Healthy aging in elderly cochlear implant recipients: a multinational observational study

**DOI:** 10.1186/s12877-020-01628-2

**Published:** 2020-07-23

**Authors:** M. Marx, I. Mosnier, J. Belmin, J. Wyss, C. Coudert-Koall, A. Ramos, R. Manrique Huarte, R. Khnifes, O. Hilly, A. Martini, D. Cuda

**Affiliations:** 1 Otology and Neurotology Department, ENT Department, Bâtiment Pierre Paul Riquet - Hôpital Purpan, Place du Dr Baylac, 31059 Toulouse Cedex 9, France; 2 Groupe Hospitalier de la Pitié-Salpétrière, Paris, France; 3 Université Pierre and Marie Curie and Hôpital Charles Foix, Paris, France; 4 Cochlear Ltd., Sydney, Australia; 5 Cochlear AG, Basel, Switzerland; 6 Complejo Hospitalario Universitario Insular Materno Infantil, Las Palmas de Gran Canaria, Spain; 7 Clinica Universitaria de Navarra, Pamplona, Spain; 8 Bnai Zion Medical Center, Haifa, Israel; 9 ENT Department at Rabin Medical Center (Beilinson), Petah Tikva, Israel; 10 ENT Otosurgery Department at Azienda Ospedaliera di Padova, Padova, Italy; 11 ENT Department of Ospedale Guglielmo da Saliceto, Piacenza, Italy

**Keywords:** Cochlear implant, Data logging, Hearing-related healthy aging, Hearing loss in elderly

## Abstract

**Background:**

Given an increase in the aging population and its impact on healthcare systems, policy makers for provision of health and social services are aiming to keep older adults in good health for longer, in other words towards ‘healthy aging’. Our study objective is to show that rehabilitation with cochlear implant treatment in the elderly with hearing impairment improves the overall health-related quality of life and general well-being that translate into healthy aging.

**Methods:**

The multicentre, prospective, repeated measures, single-subject, clinical observational study will accrue 100 elderly, first-time, unilateral CI recipients (≥ 60 years) and analyze changes on specific measurement tools over ca. 20 months from preimplant to postimplant. Evaluations will consist of details collected through case history and interview questionnaires by clinicians, data logging, self-report questionnaires completed by the recipients and a series of commonly used audiometric measures and geriatric assessment tools. The primary indicator of changes in overall quality of life will be the HUI-3.

**Discussion:**

The protocol is designed to make use of measurement tools that have already been applied to the hearing-impaired population in order to compare effects of CI rehabilitation in adults immediately before their implantation, (pre-implant) and after gaining 1–1.5 years of experience (post-implant). The broad approach will lead to a greater understanding of how useful hearing impacts the quality of life in elderly individuals, and thus improves potentials for healthy aging. Outcomes will be described and analyzed in detail.

**Trial registration:**

This research has been registered in ClinicalTrials.gov (http://www.clinicaltrials.gov/), 7 March 2017 under the n° NCT03072862.

## Background

Worldwide demographic shifts indicate that the burden of an aging population on healthcare systems will be affected by the number of babies being born into the more industrialized populations. That can be illustrated by census findings that reveal both Europe and Asia have the largest number of individuals over the age of 65 but the lowest birthrates. The US is also approaching a similar demographic. This means that the contribution to tax and social systems that fund healthcare supplied by working-age individuals will not be able to contribute enough to help finance the healthcare needs of the elderly because those contributing will be too few [58]. The PRB further reports that by 2050, individuals older than 80 years of age could reach as many as 20% of the European population. The challenge, then, is to provide means and methods that improve the health conditions of the elderly enabling them to be gainfully active as healthy-aging individuals. Utilization of effective hearing capacity may contribute to an individuals’ general health status.

In terms of healthy aging, defined by WHO (World Health Organization) as “The process of developing and maintaining functional ability that enables wellbeing in older age” [46], simply living longer is not a successful endpoint; it must include the quality of life (QoL) during a person’s extended longevity [6]. Dalton et al. [17] were one of the earliest researchers to point out specifically that hearing loss greatly affects quality of life in the elderly. We now understand that it may have a cascading effect [8] and interact negatively [6, 23, 55] with physical, cognitive and psychosocial conditions.

The presence of a HL and its considerable effects on quality of life and healthy aging has been established in various epidemiological studies [18, 27, 43], reviews [4, 10, 11, 69], meta-analyses [38, 45, 68]; and a large number of clinical studies. Contrera et al. [13] in their prospective study that enlisted both HA and CI users over the age of 50 years, concluded that CIs were more effective than HA with respect to QoL outcomes, also recognized by others as summarized in the review provided by Schaefer et al. [64]. Importantly, hearing loss and its associated effects are considered modifiable with today’s technologies [19, 28, 32, 52, 53]. Lin [43] provides a comprehensive overview about the pervasive influence of hearing loss on cognition and other domains.

Laureyns M. [37] estimated that in Europe alone, by 2025, 114 million citizens will be over the age of 65 years with 61 million self-reporting HL; that is, 54% of that older age group. Such an incidence significantly impacts the socioeconomic burden on societies. These data include only those who report hearing loss, but the number could be significantly larger as its insidious nature as a hidden disability usually develops slowly and may not necessarily be recognized. Individuals may choose to ignore HL as it may represent a stigma associated with getting old. Additionally, HL is considered a normal part of aging and, therefore, not worth reporting. These reasons highlight the importance, and urgency, to uncover and confirm the most promising methods and techniques that support healthy aging, especially by addressing currently available treatments that may modify debilitating effects such as HL.

The most common path to remediating hearing loss is through evaluations that may lead to the use of hearing aids (HA). However, in cases where HL cannot be compensated by hearing aids, cochlear implant (CI) may be a viable option [63]; however, due to financial constraints, CIs may not always be available. Thus, already in 2017, in a report called Action for Hearing Loss, WHO urged international investment in the identification and treatment of HL enabling improved access to cochlear implants, among other auditory enhancement technologies [74]. There appears to be no age limit to receiving a CI [66, 75]. The oldest recipient, to our knowledge, to receive a Nucleus/Cochlear CI was 102 years old.

With respect to benefits from cochlear implantation, strong supporting evidence suggests that an improvement in functional hearing status via the use of cochlear implants in older adults has far-reaching effects on hearing-related quality of life and other health-related domains. Earlier studies (2002–2009) were most interested in psychosocial factors such as depression and isolation [22, 51, 57, 72]; whereas, most of the later studies (2015–2019) began investigating effects on cognition [5, 9, 12, 14, 47–49, 62, 63, 66, 73]. Routine clinical follow up shows a trend for added benefits of CI treatment upon dependency, mobility and the risk of falls [35]. It should be noted that all the aforementioned studies that performed pre-post implant analyses concluded that CI benefits were observed in all areas evaluated for the study, although elderly user’s outcomes scores may have been lower than those of comparable normal-hearing adults [12]. Outcomes from longitudinal studies conducted in an aging population suggest that CI implantation may help to maintain cognitive function and delay its decline [31, 42, 48].

Benefits associated with quality of life as a primary aim of investigation have been studied [22]. was the only study to report solely on QoL, while other researchers considered additional areas that contribute to QoL [7, 73]. The current study, to our knowledge, is the only international prospective investigation currently researching the primary impact on quality of life of cochlear implantation. A similar study underway in Australia [63], aiming for 5-year follow up, has the primary goal of examining cognitive change associated with CI rehabilitation and utilizes a very different test battery with only two evaluation tools in common. The first report on outcomes confirms benefit in all areas for an initial cohort of 20 CI users with 18 month’s listening experience.

Here, we describe the study design of a multicenter prospective study in a large cohort of elderly individuals with equivalent CI experience. As to its unique features, it utilizes a full array of tests that include assessments in the physical, mental, psychosocial domains but with the primary focus on QoL.

## Methods and design

### Study aims

The primary aim is to identify significant improvement in the overall health-related quality of their life compared to their preimplant condition as measured by the HUI-3. The secondary aim is to investigate the influence of cochlear implant use on a variety of healthy-aging domains and, therefore, the overall well-being of elderly individuals. Specific healthy-aging domains to be investigated are hearing ability and communication, dependency, cognition, risk of falls, loneliness and depression/mood. Further, the study considers the impact of consistent daily use of CI communication through evaluation of data-logging outcomes.

### Study design

A repeated measure, single-subject design assesses the changes in health-related quality of life and overall well-being as the primary end-point of the study. As an observational study, no additional intervention is applied to our specific population of CI recipients. The study design is multicentre and includes language-appropriate materials relative to the participating CI recipient and has been registered at Clinicaltrials.gov identifier: NCT03072862.

### Timing schedule

**Fig. 1:**
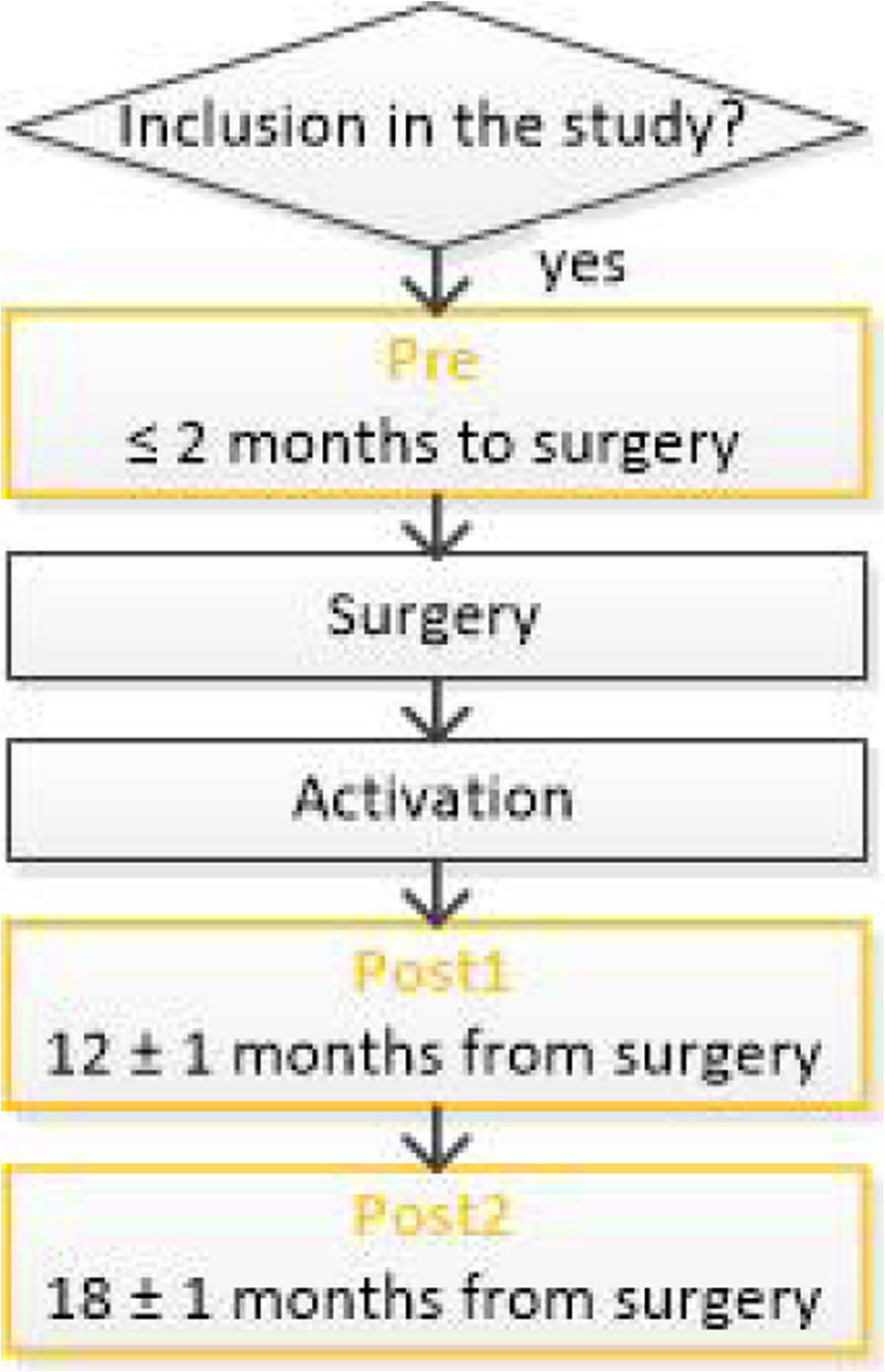
Overview of evaluation schedule flow and visits for each enrolled subject

The evaluation protocol is designed to coincide with routine clinical visits. Each subject will be assessed with the full battery of tests that are repeated at each of the three visits (Pre1, Post1, Post2, see Fig. 1). The maximum administration time for completion of all measures is estimated to be approximately two hours of which one hour is dedicated to standard self-report forms completed by the CI recipient.

### Subjects

Study participants who have agreed to a first-time unilateral implant will receive an appropriately-chosen commercially available Nucleus® Cochlear Implant Systems (Cochlear Ltd., Sydney, Australia) with the most recent implant technologies that are compatible with processors offering data logging. Subjects are aged ≥60 years with hearing loss that meets all local criteria for CI treatment. Subjects are enrolled into the clinical investigation only after signing the Patient Informed Consent Form prior to the pre-implant assessment (baseline, V1). The expected duration for each subject from enrolment is 20 months (+/− 1 month). The study aims to accrue participants distributed across all study sites and languages to avoid site and cultural bias. A maximum of 100 individuals are to be included.

Selection Criteria:

Inclusion:

- Unilateral CI candidates with bilateral postlingual deafness with intention to treat
- Contralateral ear: average pure tone thresholds indicate a moderately-severe to profound hearing loss (4 freq. Average: 0.5, 1, 2 and 3 or 4 kHz > 56 dBHL).
- Willingness to participate in and to comply with all study procedures
- Fluency in languages used to assess clinical performance
- Appropriate expectations from routine CI treatment
- Able to decide on study participation personally, and independently sign their consent

Exclusion:

- Significantly/severely dependent or fragile
- Unable to provide consent personally
- Unable to complete questionnaires for self-assessment independently
- Unilateral hearing loss
- Recipients of sequential and simultaneous bilateral CI
- Ossification or other cochlear anomalies preventing full electrode insertion or medical contraindications to surgery
- Retro cochlear or central origins of hearing impairment
- Significant comorbidities preventing study participation (e.g. blindness, immobility or in a wheel chair, severe aphasia, and other)
- Clinic Standard fail criteria for CI candidacy in regards to chronic depression, dementia, and cognitive disorders
- Unrealistic expectations on the part of the subject regarding the possible benefits, risks and limitations inherent to the procedure and prosthetic device.

### Materials

Demographic and hearing impairment characteristics, such as the aetiology of deafness, the duration of profound hearing loss, the hearing aid use, will be collected. The education level will be assessed using the ISCED (International Standard Classification of Education). In addition to these, general healthcare and patient-profile data and a selection of observational clinical assessment tools were chosen for repeated evaluations of the overall health status of the elderly individual at pre- and post-CI treatment intervals. The measures are commonly used in audiology and/or geriatric practices and are administered in the appropriate language. Translations of questionnaires have been controlled for via a validated translation process thus enabling collation of the data gathered cross culturally.

A short description of assessment tools administered at baseline are shown in Table 1 and listed alphabetically, below. The whole evaluation is performed in the subject’s individual daily listening condition, typically with unilateral or bilateral optimally fitted hearing aids before cochlear implantation, and with the cochlear implant plus a contralateral hearing aid when residual hearing in the non-implanted ear can be optimized, after the procedure.

**Table 1:** Healthy-aging domains and assessment tools used for evaluation

Domain	Clinician report			Self-report by recipient				Routine Audiology				Routine Geriatric				Device Usage
	CAP-II	L-IADL	DeJong	HUI-3	GDS-15	HHIE-S	SSQ	PTA	SFT	Sp-Q	Sp-N	DDST	MMSE	Trail B	TUG	Data Logging
Hearing	♦					♦		♦	♦	♦	♦					
Communication	♦			♦		♦	♦									♦ situations
Risk of Falls															♦	
Cognition				♦								♦	♦	♦		
Loneliness /Social Isolation			♦				♦									
Dependency		♦														
Depression/Mood			♦	♦	♦											
General Health				♦												
Device Use																♦ time

### Audiometric assessments (routine)

These include standard unaided (dB HL) and aided (dB SPL) threshold measures for frequencies 250–4000 Hz and speech discrimination in quiet and noise.

**Categories of Auditory Perception II (CAP-II)** is an auditory skill rating index consisting of nine hierarchical categories completed by the clinician as an observation of the individuals hearing abilities. The auditory skills increase in complexity ranging from perception of environmental sounds to telephone conversation with an unfamiliar speaker [25].

**Data Logging:** Cochlear sound processors feature an in-built data-logging function. Specifics of the data to be collected, along with anonymous identifying reference, include the average time on-air per day and average times in noise, speech in noise, speech, quiet, music and wind; i.e. product usage data including the use of product features and acoustic environments [60].

**De Jong Loneliness** is the six-item validated version [20] with three questions assessing social isolation and three on emotional loneliness. The scale is completed via interview.

**Digit-Symbol Substitution Test (DSST)** is pencil and paper test in which the subject is given a key grid of numbers and matching symbols (e.g. 1/−, 2/ ┴ ... 7/Λ, 8/X, 9/=) and a test section with numbers and empty boxes. The task is to fill-in as many empty boxes as possible with a symbol matching each number. The score is the number of correct number-symbol matches achieved in 120 s.

**Geriatric Depression Scale-15 (GDS-15)** is a self-administered questionnaire to record symptoms of depression in older adults. For each of the 15 questions of the scale, the subjects answer Yes or No, where ten questions indicate the presence of depression when answered positively while the other five are indicative of depression when answered negatively [65].

**Hearing Handicap Inventory in the Elderly Screening test (HHIE-S)** is a short form, self-assessment scale designed to indicate the effects of hearing impairment on emotional and social adjustment in everyday life of the elderly individual [50].

**Health Utility Mark III (HUI3)** is applied to assess health-related quality of life and is completed by the recipient. It considers, in this case, the impact of CI treatment across eight health domains (vision, hearing, speech, ambulation, dexterity, emotion, cognition and pain) [21].

**Lawton Instrumental Activities of Daily Living Scale (L-IADL)** is completed by the clinician mainly to determine a person’s ability to care for him or herself without any help from others. It measures the functional impact of emotional, cognitive, and physical impairments and their need for personal care services [39].

**Mini Mental State Examination (MMSE)-Short form-** was derived from the six memory domains of the full MMSE. It can be used to estimate the severity and progression of cognitive impairment and cognitive change in an individual over time [29].

**Speech Spatial Qualities (SSQ)** is a self-assessment scale of hearing ability and communication in daily environments, completed by the patient. It is divided into the three subcategories of speech (comprehension), spatial (hearing in space) and quality (speech and sounds) [24].

**The Timed Up and Go test (TUG)** measures the time a person takes to stand up from a standard armchair, walk three meters, turn around, walk back to the chair and then sit down again [56].

The **Trail Test B (TMT-B)** is neuro physiological test assessing executive function. It is a more cognitively demanding task than the Trail A test [70].

### Monitoring the study

The study is monitored by an external clinical research organization. A clear data management program has been set into place to validate and verify electronic data capture. Study sites and documentation may be subject to Quality Assurance audits during the course of the observational study. In addition, regulatory bodies, at their discretion, may conduct inspections, during and after study completion.

### Statistical considerations

For primary and secondary study objectives, an intra subject endpoint comparison is used: All pairwise comparisons are of interest; i.e. preimplant (Pre)-to-12 months postimplant (Post1), Pre-to-18 months postimplant (Post2), and the change from Post 1 to Post 2.

### Sample size calculation

The HUI3 will serve as the primary outcome measure for this study to determine the added health utility gain for the multi- attribute health status between Pre, Post1 and Post2. A one-way ANOVA will be performed on HUI3 multi-attribute scores, with visit as the main factor. Post-hoc tests will be performed to determine significant differences between visits.

In [71] the authors suggest that a gain of 0.1 in HUI3 multi attribute score is considered highly clinically significant. In addition, the UKCISG paper gives data on the variation in HUI3 gain across age groups receiving cochlear implants: the 95% confidence intervals for gain in HUI3 allow us to estimate the standard deviation of change of subjects aged 60+ as 0.25. Assuming a paired t-test of the difference in HUI3 multi attribute score, then with 90% power and a 5% level of significance, a mean difference of 0.1 would require 68 participants based on a standard deviation of change equal to 0.25. Because all three pairwise tests between visits are of interest Bonferroni corrections will be applied. The recomputed sample size required with this significance level requires at least 88 participants to find a change of 0.1 units in the HUI3 significance. Taking attrition into account, the inclusion of 100 study participants was deemed appropriate.

## Discussion

Our study objective is to show the potential for positive effects of CI treatment according to local indication criteria on overall health-related quality of life and general well-being that ultimately translate into healthy aging. The study outcomes are intended to provide transparent and comparable, evidence-based information to healthcare policy makers in support of guiding informed decisions on the provision of health services for the treatment of hearing loss.

In 2014, Prince et al. [59] published a report in Lancet on the leading contributors to the burden of disease in people aged > 60 years; in particular, mental and neurological disorders. Burden of disease in the aging population has a high economic impact for society. Therefore, we may assume that through CI treatment for permanent deafness, there is a potential to decrease the burden of disease in aging adults by improving important social, health and cognitive functions in addition to restoring hearing function.

The comprehensive protocol considers a range of widely accepted, interrelated metrics associated with aging beyond functional hearing. Use of this protocol does not propose to investigate causal effects but rather the pre-post implant status of CI candidates who have 1–1.5 years of experience using their unilateral implants. In line with existing study designs for CI recipients, we have chosen both self-report and clinician-report questionnaires that are essentially non-verbal, thus removing challenges directly associated with utilizing newly acquired functional hearing [32]. All the standardized tests have been applied to CI recipients in published research with the exception of the Lawton iADL and the DSST both of which have been used in assessing hearing-impaired individuals with hearing aids [3, 43].

The HUI-3, as the primary instrument in assessing CI implant treatment effects on overall health-related quality of life of elderly individuals, has been recommended as the most sensitive and useful tool for CI users [1, 16, 34, 61]. General functional hearing is addressed by audiometric evaluations and the CAP-II, which is most often used in a younger hearing-impaired population. However, it has also been applied to adult CI users [54, 72].

Further, the protocol investigates the major categories of psychosocial, mental, physical and communication behaviors with a range of other assessment tools currently applied to hearing-impaired individuals. The psychosocial domain regarding the issue of depression has been measured by the GDS-15 in CI users [9, 57]. Dependency and loneliness are assessed by the deJong Loneliness Scale that has yielded results in studies of CI users [7] and the Lawton in HA users [3]. Cognition is evaluated with the MMSE in CI studies [36, 47, 48, 62] and the Trail B by [49, 73] in CI. The Digit-Symbol has been applied to HA users [43] but it has also been recommended for CI users [30]. The physical domain focuses on the tendency to fall and is addressed by the TUG applied to CI users [2, 40]. Finally, general communication is addressed by the SSQ previously used in CI users [41, 51, 76], and the HHIE. The HHIE was included as it is one of the most widely applied questionnaires with respect to auditory participation [67] and has been reported with CI users [44].

Data logging is an important addition as it may provide new insights and potential correlations with healthy-aging domains. As has been pointed out by [4], there have been no studies, to our knowledge, investigating the impact of CI and their relationship to complex social environments. Data logging may serve as an initial step by enabling comparison of different sound environments (scenes), especially quiet versus noise [15]. A further advantage of data logging is use time. Several authors have posited that hours of auditory input or CI use per day have a significant effect on socialization and other psychosocial outcomes [26, 33]. Although data logging first became available in 2013, no studies have correlated outcomes with impact on quality of life.

A potential limitation of the study is the lack of a control group. In fact, establishing a randomized-controlled study design is not straightforward as it would imply the need not to give a CI to those control patients who meet criteria for cochlear implantation, which is far from ethical. The single-subject, repeated-measures design allows for subjects to serve as their own controls, and thus increases statistical power. But, the lack of preoperative repeated measures does not allow an estimate of within-subject variations in a non-intervention state. To counter this, the relatively large population size allows for a good estimate of effect size and would make the results broadly applicable. Another limitation is that the investigator or their team may have access to preoperative evaluation information at the time of the follow-up evaluations. However, many of the test results require coding in order to obtain a score and places a layer of protection against investigators making comparisons at the time of evaluation.

Ultimately, the study aim is to provide clinical evidence that CI treatment in aging adults currently has the potential to improve the overall health status including, but not exclusive to, hearing function, which in turn can create cost savings from a payer and societal perspective. Age-related HL is often ignored as a stigma associated with aging or just accepted as a natural side-effect of it. Therefore, growing evidence may remove these boundaries and support referring professionals and potential CI candidates in their decision for CI treatment as soon as significant hearing loss is diagnosed in the hopes of reducing the overall impact of hearing disability.

## Data Availability

Data generated from the current study will be made available to study investigators on reasonable request. Study investigators may publish their local data separately. Overall outcomes of the study are intended for publication in peer-reviewed journals and proceedings, professional forums, international presentations and lectures and other dedicated communications. As well as grouped findings, each participating clinic may choose to disseminate their own data via local media.

## Abbreviations

CAP-II: Categories of Auditory Perception II
CI: Cochlear implant
DSST-Digit: Symbol Substitution Test
GDS-15: Geriatric Depression Scale-15
HA: Hearing aids
HHIE-S: Hearing Handicap Inventory in the Elderly Screening test
L-IADL: Lawton Instrumental Activities of Daily Living Scale
MMSE: Mini Mental State Examination
PRB: Population Reference Bureau
PTA: Pure tone Audiometry
QoL: Quality of life
SFT: Sound Field Thresholds
SSQ: Speech Spatial Qualities
TMT-B: Trail Test B
TUG: The Timed Up and Go test
WHO: World Health Organization
